# Research advances in serum chitinase-3-like protein 1 in liver fibrosis

**DOI:** 10.3389/fmed.2024.1372434

**Published:** 2024-06-19

**Authors:** Xingwei Hu, Wenhan Liu, Jianhua Liu, Bojian Wang, Xiaosong Qin

**Affiliations:** ^1^Department of Laboratory Medicine, Shengjing Hospital of China Medical University, Shenyang, Liaoning, China; ^2^Liaoning Clinical Research Center for Laboratory Medicine, Shenyang, Liaoning, China

**Keywords:** biomarker, chitinase-3-like protein 1, liver fibrosis, non-alcoholic fatty liver disease, viral hepatitis

## Abstract

While liver fibrosis remains a serious, progressive, chronic liver disease, and factors causing damage persist, liver fibrosis may develop into cirrhosis and liver cancer. However, short-term liver fibrosis is reversible. Therefore, an early diagnosis of liver fibrosis in the reversible transition phase is important for effective treatment of liver diseases. Chitinase-3-like protein 1 (CHI3L1), an inflammatory response factor that participates in various biological processes and is abundant in liver tissue, holds promise as a potential biomarker for liver diseases. Here, we aimed to review research developments regarding serum CHI3L1 in relation to the pathophysiology and diagnosis of liver fibrosis of various etiologies, providing a reference for the diagnosis, treatment, and prognosis of liver diseases.

## 1 Introduction

Liver fibrosis, caused by etiological factors such as hepatitis viruses, non-alcoholic fatty liver disease (NAFLD)/non-alcoholic steatohepatitis (NASH), toxins, and alcohol consumption, is a key driver of various chronic liver diseases and cirrhosis and is strongly associated with the prognosis of chronic liver disease (1–3). Liver fibrosis is a wound-healing response against hepatocyte injury. During liver fibrosis, extracellular matrix (ECM; rich in collagen I and III) formation increases, and hepatic stellate cells (HSCs) gradually transform into myofibroblasts, ultimately leading to a reduction in hepatocytes and an accumulation of ECM and fibrillar collagen (4). Liver fibrosis is histologically reversible, whereas cirrhosis reversal is more challenging (5). Therefore, early diagnosis of liver fibrosis and effective treatment during its reversible period are crucial.

Histopathological assessment remains the gold standard for diagnosing liver fibrosis Histopathological assessment provides key findings for a definitive diagnosis, allowing for the measurement of inflammatory activity, the degree of fibrosis, and the determination of therapeutic efficacy. However, as an invasive test, a liver biopsy carries a risk of postoperative complications such as pain, bleeding, infection, and even death. It is a costly procedure and may not always be well tolerated by patients. Moreover, the scope of liver biopsy is further limited in terms of sampling and inter-observer errors, highlighting limitations in accurately diagnosing and periodically evaluating the degree of liver fibrosis. Non-invasive imaging techniques, including transient elastography (TE), ultrasound radiation force impulse imaging (ARFI), and magnetic resonance elastography (MRE), are frequently used as liver stiffness measurement (LSM) tests. However, since the M-probe can only detect a depth of 2.5–6.5 cm below the hepatic pericardium, obese patients may not obtain sufficient signals. There are also limitations for patients with ascites around the liver because shear waves cannot propagate in fluid. Therefore, TE cannot be used in patients with ascites or obesity (6). In addition, its results vary according to the experience of the operator, and differentiating between progressive and significant hepatic fibrosis can be challenging. While the overall efficacy of MRE is superior to that of TE, there are currently no guidelines concerning diagnostic thresholds for liver fibrosis that incorporate MRE-related liver elasticity values. Moreover, MRE is not a substitute for liver biopsy.

Serologic indicators provide unique advantages as they are non-invasive, simple, and highly reproducible. In addition, compared to biopsy testing, these markers offer the added advantage of indicating the level of fibrotic changes occurring throughout the liver. Determining the extent of liver fibrosis sensitively and accurately is difficult using only a single index, and in recent years, many non-invasive diagnostic models have been developed to partially replace liver biopsy. Currently, the commonly used serological diagnostic models are four serum liver fibrosis markers, namely, amino-terminal pro-peptide of type III pro-collagen (PIIINP), collagen IV (CIV), laminin (LN), and hyaluronic acid; the fibrosis-4 (FIB-4) index (7); and the aspartate aminotransferase (ASP)-to-platelet ratio index (APRI) (8). FIB-4 and APRI reduce the need for a liver biopsy by approximately 30–40%. The 2015 World Health Organization (WHO) guidelines for the prevention and treatment of hepatitis B and the Chinese guidelines for the prevention and treatment of chronic hepatitis B (CHB) both recommend FIB-4 and APRI, for the assessment and diagnosis of liver fibrosis. However, the diagnostic value of FIB-4 and APRI is limited to patients with CHB and chronic hepatitis C (CHC), and the diagnostic value of these serological markers in determining intermediate stages of liver fibrosis, specifically in diagnosing liver fibrosis due to other causes, remains unclear (9) (Table 1). CHI3L1, a member of the chitosanase family, is highly expressed in liver tissues, can be secreted into the ECM of the liver, and is highly expressed in hepatic fibrosis. Therefore, by reviewing the recent developments in serum CHI3L1 research, that is, the role of CHI3L1 in the pathogenesis and diagnosis of liver fibrosis, caused by different etiologic factors, we aimed to determine reference values to aid in the diagnosis, treatment, and prognosis of liver diseases.

**Table 1:** Diagnostic value of serum markers for liver fibrosis.

Index	Parameters	Diseases (cases)		AUC	Sensitivity (%)	Specificity (%)	Negative predictive value (%)	Positive predictive value (%)	REF
CHI3L1		CHB	Monoinfected with HBV	0.97	95.20	89.70			(10)
			HBeAg-negative patients with CHB	0.818	80.00	71.05			(11)
			Significant fibrosis (≥F2)	0.728	59.1	75.6			(12)
		CHC		0.809	78	81			(13)
		NAFLD		0.7638	70	76.80			(14)
APRI	AST, PLT	CHB		0.688	55.20	85.70			(10)
		CHC		0.8	91	47			(8)
		NAFLD		0.77	18.30	96.10			(6)
		ALD		0.7	13.20	77.60			(15)
FIB-4	PLT, ALT, AST, age	CHB		0.7844	65.40	73.60			(16)
		NAFLD		0.85	84.40	68.50			(17)
		ALD		0.85	58	91			(18)
PP score	PLT, PIIINP	Early treatment stage for CHB	Advanced fibrosis (*F* ≥ 3, 225 cases)	0.775					(9)
			Advanced fibrosis (*F* ≥ 4, 117 cases)	0.803					
			Liver cirrhosis (*F* ≥ 5, 29 cases)	0.803					
		Post-treatment CHB	Advanced fibrosis (F ≥ 3,137 cases)	0.632					
			Advanced fibrosis (F ≥ 4, 65 cases)	0.700					
			Liver cirrhosis (F ≥ 5, 21 cases)	0.743					
NIS4 score	miR-34a-5p, A2M, CHI3L1, HbA1c	Prevalence of at-risk NASH*	Pooled validation cohort (*n* = 702)	0.80	81.50	63.00	77.90		(19)
CAP	CHI3L1, AFP, PLT	CHB, 337 cases		0.805–0.819	71.60–81.30	70.00–79.89			(20)
YKL-40 model	CHI3L1, AST, HA, PLT	ALT<2x the ULN CHB	Training group, 307 cases	0.786	71.74	72.85	80.88	61.68	(21)
			Validation group (153 cases)	0.831	71.79	85.33	85.33	71.79	

AUC, area under curve; REF, references; CHB, chronic hepatitis B; CHC, chronic hepatitis C; NAFLD, non-alcoholic fatty liver disease; NASH, non-alcoholic steatohepatitis; ALD, alcoholic liver disease; ULN, upper limit of normal.

*At-risk NASH was defined as non-alcoholic fatty liver disease activity score 4 or more and liver fibrosis stage 2 or more.

## 2 Overview of CHI3L1

### 2.1 CHI3L1 structure and receptors

CHI3L1, commonly known as the chitin protein, is a glycoprotein (with a molecular weight of approximately 40 kDa) expressed by the *CHI3L1* gene located on chromosome 1q31-q32.35. CHI3L1 belongs to the 18-glycosyl hydrolase family, and its polypeptide chain consists of 383 amino acids. It has been named YKL-40, and owing to the N-terminal three amino acids at the N-terminal end of the peptide chain are tyrosine (Y), lysine (K), and leucine (L). The 18-glycosyl hydrolase family mainly includes chitinases and chitinase-like proteins (CLPs). Chitinases are proteins with true chitin-degrading ability, while CHI3L1 belongs to the CLPs. Due to a mutation in the catalytic residue glutamate, CHI3L1 does not possess hydrolase activity; however, it still has a high affinity for chitosan, binds to a variety of receptors, and induces a wide range of cellular responses (22, 23). Currently identified CHI3L1 receptors include heparin, collagen, IL-13Rα2, transmembrane protein 219 (TMEM219), galectin-3, and cluster of differentiation 44 (CD44). CHI3L1 functions by binding to the receptors.

For example, CHI3L1binds to CD44 and activates the ERK and Akt pathways, as well as phosphorylates β-catenin, which promotes metastasis in gastric cancer (24). In addition, CHI3L1 modulates the glioma microenvironment by interacting with galectin-3, increasing tumor immunosuppression, and promoting macrophage M2 polarization, a process that is negatively regulated by galectin-3-binding proteins by competing with galectin-3 for binding to CHI3L1 (25).

### 2.2 Synthesis of CHI3L1

CHI3L1 is a highly evolutionarily conserved secreted protein, first discovered in 1992 by Johansen et al. in the cell culture of the human osteosarcoma cell line, MG63 (26). Immunofluorescence staining of liver specimens from patients with NAFLD revealed that CHI3L1 in liver tissue was mainly derived from macrophages. Studies have shown that CHI3L1 is also derived from a variety of cells, including neutrophils, fibroblasts, vascular smooth muscle cells, chondrocytes, HSCs, and tumor cells (27–29).

CHI3L1 synthesis and secretion are regulated by a variety of factors. Many cytokines such as IL-1β, IL-13, IL-6, and IFN-γ stimulate CHI3L1 expression, which in turn can regulate the expression of cytokines such as IL-6, IL-8, IL-12, IL-18, IFN-γ, and tumor necrosis factor (30). Non-coding RNAs such as miR-125-3p, miR-342-3p, and linc00963 regulate CHI3L1 through signaling pathways and play a key role in regulating inflammation-driven liver fibrosis (31–33). Sarma et al. (32) reported that CHI3L1 expression is regulated through the miRNA-449a/NOTCH1 axis and that stabilized p65 interacts with CCAAT/EBPα in the CHI3L1 promoter region to upregulate CHI3L1 expression in hepatitis C. Furthermore, factors such as aging, ECM changes, stress, and drugs also modulate CHI3L1 expression (34).

### 2.3 Biological functions of CHI3L1

**Figure 1:**
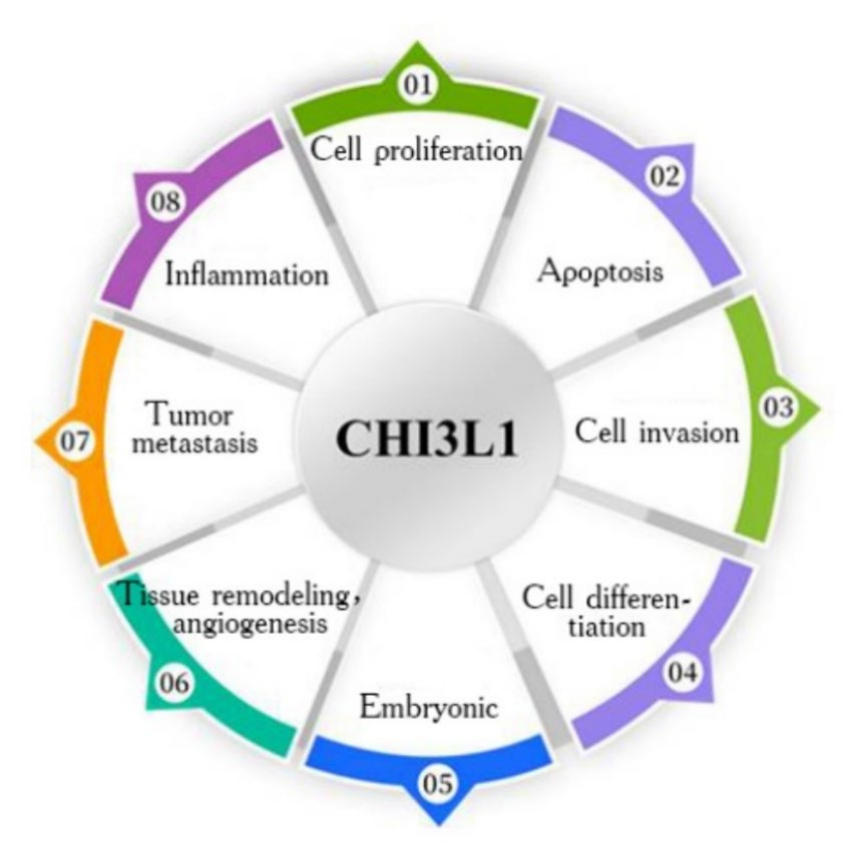
Biological functions of CHI3L1.

CHI3L1 is closely related to cell proliferation, apoptosis, cell differentiation, and cell invasion and is involved in embryonic development, inflammation, tissue remodeling, angiogenesis, and tumor metastasis (Figure 1); however, studies targeting serum CHI3L1 levels are limited (35). Additionally, CHI3L1 is involved in innate immune system and ECM remodeling and is associated with chronic viral hepatitis, alcoholic hepatitis, NAFLD, and other chronic liver diseases, with a close association between the degree of liver fibrosis and ECM synthesis (14, 36–38).

#### 2.3.1 CHI3L1 as an inflammatory factor

Infection and inflammation stimulate the production of CHI3L1, which plays a major role in tissue injury, inflammation, tissue repair, and remodeling responses. CHI3L1 expression is upregulated in inflammatory conditions such as ulcerative colitis, Crohn’s disease, rheumatoid arthritis, osteoarthritis, and hepatic sclerosis, as well as in solid cancers (22, 39–41). CHI3L1plays a key role in microenvironmental remodeling in different diseases (42). Schoneveld et al. (43) retrospectively analyzed serum CHI3L1 levels in patients with coronavirus disease 2019 (COVID-19), chronic obstructive pulmonary disease, and unrelated interstitial lung disease and found that CHI3L1 upregulation in patients was strongly associated with the level of inflammation. De Lorenzo et al. (44) found that plasma CHI3L1 levels were elevated in patients with COVID-19 and that this elevation might contribute to a poor prognosis. The researchers also reported that although systemic inflammation had returned to baseline levels and plasma CHI3L1 levels decreased after 4 weeks of clinical remission in the patients with COVID-19,CHI3L1 levels in individuals that recently presented with systemic inflammation were significantly higher than those in healthy controls. This suggests that even after systemic inflammation returns to baseline levels, the stimuli that maintain CHI3L1 production may still be present.

#### 2.3.2 Immunosuppressive effects

CHI3L1 has immunosuppressive functions and has been identified as a new target for T-cell blockade (45, 46). CHI3L1 blocks T-cell infiltration by promoting neutrophil recruitment and neutrophil extracellular trap formation, and CHI3L1 targeting promotes antitumor immunity in various tumor types (47). For example, in glioblastoma, upregulation of cancer-intrinsic CHI3L1 signaling regulates the immunosuppressive microenvironment by reprogramming tumor-associated macrophages, leading to tumor progression (25). CHI3L1 deficiency accelerates stroke by enhancing neuroinflammation via reduced M2 macrophage polarization (48–50). NOD-like receptor protein 3 (NLRP3) mimics some of the hepatic features of NASH, and breast regression protein 39 (BRP39) plays a regulatory role in NLRP3-mediated hepatic inflammation and fibrosis in NLRP3 hyperfunctional mice. f BRP39 knockdown reduces hepatic inflammation and fibrosis and decreases infiltrating lipid-associated macrophages and neutrophils, two immune cells that play a key role in NASH progression (51). These findings suggest that CHI3L1 may be a novel target for the treatment of immune liver injury as well as for other T-cell-mediated diseases.

## 3 The relationship between CHI3L1 and liver fibrosis

Studies have shown that a variety of cells, including myofibroblasts, HSCs, hepatocytes, inflammatory cells, liver sinusoidal endothelial cells (LSECs), portal fibroblasts, and fibrocytes, are involved in liver fibrosis. Myofibroblasts are the main source of ECM in fibrotic liver and are not present in healthy liver tissues. Activated HSCs and portal fibroblasts transform into myofibroblasts during fibrosis, where HSCs play an important role in hepatic fibrosis. Quiescent HSCs reside in the defined space between LSECs and hepatocyte clusters (space of Disse). Upon activation, HSCs migrate to the site of injury and secrete ECM. CHI3L1 has been identified as a pro-fibrotic factor; it is overexpressed in aging livers and in patients with hepatic cirrhosis, thus given its key role in the development of hepatic fibrosis through direct action on HSCs and promotion of susceptibility to fibrosis in aging livers (34), CHI3L1 serves as a biomarker of hepatic fibrosis.

In a previous study involving 168 patients (non-hepatitis B, *n* = 79 patients; hepatitis B, *n* = 89 patients), we observed that in both patients with CHB and those without, there was a statistically significant difference in serum CHI3L1 levels between the significant and non-significant hepatic fibrosis group (Tables 2, 3; Supplementary material). It has been shown that in human and mouse fibrotic livers, CHI3L1 is mainly derived from hepatic macrophages and the accumulation of CHI3L1-positive hepatic macrophages is markedly enhanced during the fibrotic process, which may account for the elevated serum CHI3L1 levels (52). In a NASH mouse model, CHI3L1 regulated macrophage-hepatic stellate cell crosstalk, and direct stimulation of macrophages by CHI3L1 led to the upregulation of HSC-activating factor expression. In an *in vitro* study, stimulation of LX-2 cells with recombinant CHI3L1 showed direct activation of HSCs by CHI3L1 through the receptor IL13Rα2, which led to the upregulation of pro-fibrotic factors in the liver (53). Recombinant CHI3L1 promotes the proliferation and activation of primary human HSCs (34).

**Table 2:** Serum CHI3L1 levels in patients with CHB.

Patients with CHB	M (P25, P75)	Median difference (95% CI)	Wilcoxon two-sample rank sum test	
			*Z-*value	*p*-value
No significant liver fibrosis group (*n* = 45)	95.18 (68.39–112.38)	−73.489 (−87.320 to −59.509)	−7.476	<0.001
Significant liver fibrosis group (*n* = 44)	165.23 (141.11–192.65)			

**Table 3:** Serum CHI3L1 levels in patients with no hepatitis B.

Patients with no CHB	M (P25, P75)	Median difference (95% CI)	Wilcoxon two-sample rank sum test	
			*Z*-value	*p*-value
No significant liver fibrosis group (*n* = 30)	113.11 (97.56–118.60)	−8.153 (−16.069 to −0.453)	−2.101	0.036
Significant liver fibrosis group (*n* = 49)	118.49 (103.62–128.68)			

CHI3L1 inhibits hepatic macrophage apoptosis by suppressing Fas expression and activating the Akt signaling pathway in an autocrine manner, leading to hepatic macrophage accumulation and activation, which exacerbates liver fibrosis. CHI3L1 inhibits apoptosis in M1-like but not M2-like hepatic macrophages (52). In conclusion, by inhibiting hepatic macrophage aggregation and promoting apoptosis, CHI3L1 deficiency could ameliorate hepatic fibrosis; thus,CHI3L1 can serve as a potential therapeutic target for liver fibrosis.

## 4 Application of CHI3L1 in the diagnosis of liver fibrosis

### 4.1 CHB

Hepatitis B virus (HBV) infection is a major global public health issue. WHO estimates that of the 296 million people chronically infected with HBV globally, nearly 820,000 died due to HBV-related diseases in 2019. Furthermore, in approximately 15–40% of untreated patients, HBV infection will progress to cirrhosis or hepatocellular carcinoma (HCC). As the world’s most populous country, China has approximately 100 million HBsAg carriers (prevalence, 7.8%), and the annual number of deaths from HBV-related liver diseases is 162,000, accounting for approximately 29% of HBV-related deaths globally (54). China has made efforts to reduce the incidence of HBV infection over the past three decades (55), and aims to be a major contributor to the WHO’s goal of “eliminating viral hepatitis as a major public health threat by 2030.”

CHB is a dynamically progressive disease, and several clinical studies have shown that timely and effective antiviral therapy can slow or even reverse CHB-induced liver fibrosis (56, 57). The American Association for the Study of Liver Diseases and Asian Pacific Association for the Study of Liver Diseases guidelines recommend antiviral therapy for patients when the alanine transaminase (ALT) levels that are twice the upper limit of normal (ULN) levels (58, 59). However, in Chinese patients with CHB, ALT levels and the degree of fibrosis are not consistent, regardless of the HBe-Antigen (HBeAg) status and HBV DNA levels. Moreover, significant hepatic fibrosis has also been present in patients with CHB and an ALT level less than twice the ULN (21). Therefore, in the 2022 Expert Opinion on Expanding Antiviral Therapy for CHB, published by the Chinese Medical Association’s Section of Hepatology, it was suggested that antiviral therapy be initiated when a non-invasive diagnosis suggests significant inflammation or fibrosis in the liver (60).

Serum CHI3L1is associated with a high diagnostic value in CHB-related liver fibrosis. In HBeAg-negative patients with CHB, serum CHI3L1 has high diagnostic efficiency in the staging of liver fibrosis, with a sensitivity and specificity of 80.00 and 71.05%, respectively (11). In patients with CHB and an ALT less than twice the ULN, serum CHI3L1 levels are independently associated with advanced liver fibrosis and serve as a potential biomarker for liver fibrosis. The study also constructed a model based on CHI3L1, which significantly outperformed the existing scores in patients with CHB having normal and mildly elevated diagnostic ALT levels (FIB-4, APRI, Huu Model, and Forns’ index). This finding may guide clinicians in diagnostically identifying patients who may benefit from antiviral therapy (21).

Huang et al. (20) used univariate and multivariate logistic regression analyses to identify three independent predictors of advanced liver fibrosis in 337 patients with CHB (CHI3L1, AFP, and PLT indexes), and a new diagnostic model was established, namely, the CHI3L1/AFP/PLT (CAP) index. This index facilitated the diagnosis of advanced liver fibrosis with an AUROC significantly higher than that in the APRI and FIB-4, which is more suitable for patients with CHB. The diagnostic efficacy in advanced liver fibrosis is not affected by ALT level and HBeAg status, making it suitable for patients with CHB at different stages and helps in predicting the timing of antiviral therapy in these patients. A research team from Ningbo University examined the serum CHI3L1 expression in patients with CHB, liver cirrhosis, and HCC and investigated the expression characteristics of chronic liver diseases related to hepatitis B in different stages. The results of the investigation showed that the expression level of CHI3L1 progressively increased from CHB and liver cirrhosis to HCC, and the CHI3L1 expression offers clinical value in evaluating the different stages of chronic liver diseases and may be used as an indicator to monitor the disease evolution (10). However, other similar studies have suggested that CHI3L1 levels cannot significantly differentiate early liver fibrosis (meta-analysis of histologic data in viral hepatitis [METAVIR]F0, F1, and F2) (12).

CHI3L1 is a useful non-invasive marker for assessing liver fibrosis prior to treatment in patients with CHB and monitoring changes in liver fibrosis during treatment. Lin et al. (38) compared serum CHI3L1 levels, the hepatic tissue collagen proportional area (CPA), and LSM in 131 patients with CHB who received entecavir antiviral therapy for 78 weeks and found that serum CHI3L1 levels decreased from baseline after 78 weeks of treatment and were positively correlated with CPA and LSM.

In conclusion, the value of CHI3L1 expression in assessing significant liver fibrosis in patients with CHB is clearer and superior to currently known non-invasive diagnostic methods, and it serve as a reliable reference for antiviral therapy. However, as the diagnosis of early stage liver fibrosis is not highly accurate, a more appropriate non-invasive indicator for an individual or combined diagnosis remains to be identified.

### 4.2 CHC

Hepatitis C infection is a global epidemic trend, and people of different sexes, ages, and ethnicities are generally susceptible to HCV. According to the WHO, approximately 58 million people worldwide are currently infected with HCV, and approximately 290,000 deaths are attributed to HCV infection annually. In 2020, China had the largest number of HCV-infected individuals (approximately 9,487,000 people) (61). HCV infection is prone to chronicity, with approximately 55–85% of cases of acute hepatitis C developing into CHC. Cirrhosis and HCC are the main causes of death in patients with CHC (62). There is no effective vaccine to prevent HCV infection; however, as the treatment of hepatitis C enters a new era in terms of direct antiviral agents (DAAs), with an increasing number of patients achieving a sustained virologic response (SVR), post-treatment benefits and clinical outcomes are fast becoming the focus for future studies. The prognosis of patients after viral clearance is closely related to the degree of fibrosis (63), the early diagnosis of HCV-associated hepatic fibrosis, and staging; thus, timely interventions are crucial.

Little is known concerning the progression of hepatic fibrosis after HCV eradication; however, recent studies have suggested that serum CHI3L1 levels may be a non-invasive marker for monitoring fibrosis in patients with CHC. Treatment with DAAs significantly and sustainably improved hepatic fibrosis in patients with CHC, with serum CHI3L1 levels being significantly lower at the end of treatment compared baseline levels (64–66). In one study involving 105 patients with CHC treated with DAAs, CHI3L1 was identified as a sensitive marker for monitoring changes in fibrosis during treatment and in the weeks after having reached an SVR. Early identification of treatment success at the end of treatment using CHI3LI levels may facilitate a timely shift to alternative treatments (64). Similar international studies have been conducted. Researchers at Helwan University studied the risk of HCC susceptibility in Egyptian patients with hepatitis C after SVR through DAA treatment and found that the *CHI3L1* gene (rs880633) could serve as a strong predictor and risk factor for patients to develop HCC post-SVR (67). While some studies have suggested that diagnosis using non-invasive indicators can be confounded by factors such as liver inflammation, a study from the First People’s Hospital of Peking University concludes the utility of treatment with DAAs to achieve a rapid and significant reversal of hepatic fibrosis in addition to relieving inflammation and highlights that this regression can be detected as early as the end of therapy (66).

In summary, serum CHI3L1 levels are associated with the severity of fibrosis in CHC and its progression over time, which is clinically important for monitoring the degree of hepatic fibrosis and thus predicting the clinical outcome in patients with an SVR after treatment with DAAs. However, the benefits of CHI3L1 in the diagnosis of mild and severe fibrosis remain unclear.

### 4.3 Chronic hepatitis D

In recent years, there has been a renewed focus on hepatitis D virus (HDV) in several countries and regions, and comprehensive reports have shown that there are approximately 15–20 million cases of HDV infection worldwide, which is equivalent to approximately 5% of the chronically infected population with HBV (68). HDV is a defective RNA virus that requires the aid of HBV to complete its life cycle. Compared with HBV infection alone, overlapping HBV/HDV infections accelerate disease progression. It has been estimated that 70% of patients infected with HDV develop cirrhosis within 5–10 years and 60% succumb to disease within 10 years, in addition to a 28% increase in the risk of HCC in those infected with HBV/HDV (69, 70).

In the process of eliminating the viral hepatitis public health threat, it is important to emphasize and act in relation to chronic HBV, HCV, and HDV infection. With the application of nucleoside (acid) analogs and polyethylene glycol interferon-alpha, CHB can be effectively controlled, resulting in HBsAg-negativity and potential reversal of hepatic fibrosis, or even re-compensation after decompensation, can be achieved in some patients. Therefore, hepatitis D is emerging as a chronic viral hepatitis that cannot be neglected in the process of eliminating viral hepatitis-related public health hazards. At present, there are few studies on the use of CHI3L1 for the diagnosis of liver fibrosis due to HBV/HDV overlapping infections. One study in the United States (34) detected *CHI3L1* gene expression in the liver tissues of 64 patients with liver cirrhosis of different etiologies using quantitative reverse transcription polymerase chain reaction testing: CHI3L1 expression was significantly higher in the livers of patients with liver cirrhosis of various etiologies than in controls, with the highest in cirrhosis due to HDV, followed by HCV, HBV, and alcoholic cirrhosis. Interestingly, CHI3L1 was significantly higher in HDV than in HBV cirrhosis, despite HDV dependence on HBV. In the study, all patients with HDV-induced liver cirrhosis tested positive for serum HDV RNA and anti-HDV (IgG), with 82% of patients positive for anti-HDV (IgM). In addition, patients with HDV liver cirrhosis had the lowest platelet counts, consistent with typical splenomegaly, and the highest activity grading (*p* < 0.001) compared with alcohol-related cirrhosis and hepatitis C- and B-related diseases, reconfirming that hepatitis D is the most severe form of chronic viral hepatitis. In conclusion, chronic HDV infection is currently underappreciated and understudied in China, and the important role of CHI3L1 in chronic HDV progression and diagnosis needs to be further explored.

### 4.4 NAFLD

Currently, NAFLD is the most common chronic liver disease worldwide, with a prevalence rate of up to 25%. It is one of the leading causes of liver disease-related mortality (71). Characterized as excessive accumulation of liver fat, NAFLD is due to hepatic steatosis without excessive alcohol consumption and is a type of metabolic stress liver injury closely related to insulin resistance and genetic susceptibility, which mainly includes NASH, cirrhosis, and HCC. Approximately 20% of patients with NAFLD can progress to more severe NASH (72, 73), a severe type of NAFLD characterized pathologically by inflammation, hepatocellular injury, lipid degeneration, and fibrosis. With type 2 diabetes and obesity, NASH is increasingly becoming a public health concern, and, without clinical intervention, it can progress to severe liver diseases such as liver failure, cirrhosis, and HCC, potentially necessitating liver transplantation (72). Among various liver histologic indices, the liver fibrosis stage is an independent predictor of long-term prognosis in patients with NAFLD. Therefore, early identification of patients with NASH and significant fibrosis is crucial.

Integration of liver transcriptome datasets using the robust rank aggregation method to construct transcriptomic profiles of NASH progression and fibrosis severity in patients with NAFLD revealed that the *CHI3L1* gene was located in the top 10 upregulated genes in patients with NASH (74). One study suggests that CHI3L1 may be a potential marker for predicting significant fibrosis in patients with NAFLD (75). Kumagai et al. (14) measured serum CHI3L1 levels in 111 patients with NAFLD and 23 patients with HCC combined with NAFLD and found that serum CHI3L1 levels in patients with NAFLD increased with the progression of hepatic fibrosis. Additionally, CHI3L1 was significantly correlated with severe fibrosis (F3–4), and patients with HCC combined with NAFLD had significantly higher serum CHI3L1 levels than patients with NAFLD and non-HCC.

Harrison et al. (19) developed and externally validated a new blood-based non-invasive diagnostic model, namely, the NIS4 score, consisting of the following four metrics: miR-34a-5p, α-2 macroglobulin, CHI3L1, and glycated hemoglobin, specifically designed to identify patients with metabolic risk factors (type 2 diabetes mellitus, obesity, dyslipidemia, and hypertension) in patients with high-risk NASH (including those with an NAFLD activity score ≥ 4 and fibrosis stage ≥2). This model offered improved NIS4 diagnostic performance and was not affected by age, sex, body mass index, or aminotransferase concentration. The study concluded that although the diagnostic efficacy of CHI3L1 alone was not significant, the NIS4 for high-risk NASH outperformed other blood-based diagnostic scores such as the FIB-4, the NAFLD fibrosis score(NFS), and APRI (NIS4, AUC = 0.80; CHI3L1, AUC = 0.69; APRI, AUC = 0.74; FIB-4, AUC = 0.70; NFS AUC = 0.66). The data also showed that while FIB-4 and NFS showed high specificity, their low sensitivities could lead to misleading diagnostic results for NASH, especially for the adjudication of high-risk NASH cases. In contrast, the sensitivity and specificity of NIS4 were optimally balanced. NFS mainly targets NAFLD-associated liver fibrosis; however, it is not a suitable screening indicator because it leads to overdiagnosis and high percentage of false negatives (76). The NIS4 model was further optimized in 2023 as the NIS2 + ™ (including miR-34a-5p and YKL-40), providing a more effective non-invasive method in which to rule out high-risk NASH for patients at risk (77).

Hepatic insulin resistance is known to have an important role in the development of NASH; however, the exact mechanism of action remains unclear. Zhang et al. (78) suggested that *CHI3L1* gene upregulation may be an important factor in the generation of the NAFLD/NASH phenotype. The researchers constructed a CHI3L1 knockout mouse model and observed improved insulin signaling in the CHI3L1 knockout mice compared with C57BL/6 wild-type (WT) mice fed the same diet, suggesting that decreasing the expression of CHI3L1 in the liver or inhibiting its function could ameliorate insulin resistance in the liver. To further confirm this finding, three anti-CHI3L1 monoclonal antibodies (FRG, CH568, and CHXI3B6) were used in the study. All three anti-CHI3L1 monoclonal antibody proteins inhibited their *in vivo* functions of CHI3L1 to varying degrees and significantly improved insulin resistance in the liver after 16 weeks of anti-CHI3L1 monoclonal antibody treatment. Additionally, although the CHI3L1 protein was expressed at different levels in the hepatocytes of patients with NAFLD and NASH, the positive cells of both were mainly localized in the hepatoportal vasculature, and immunohistochemical staining revealed abundantly expressed CHI3L1 in infiltrating inflammatory cells and hepatocytes. Overall, CHI3L1 is important for the diagnosis of advanced NAFLD-associated hepatic fibrosis and increased CHI3L1 expression directly correlates with hepatic fibrosis progression. CHI3L1 is expected to be a therapeutic target against monoclonal antibodies, but its role in the mechanism of hepatic fibrogenesis needs to be further investigated. Future studies are required to confirm the role of CHI3L1 in hepatic fibrosis (that is, determine whether CHI3L1 is a co-existing hepatic fibrosis factor or whether it promotes the formation of hepatic fibrosis).

### 4.5 Other liver diseases

In addition to viral hepatitis and NAFLD, excessive alcohol consumption and autoimmune liver disease can lead to serious negative outcomes. Among the different types of fatty liver diseases, the incidence of alcohol-related liver disease(ALD) related cirrhosis and HCC is the highest (79). However, the main cause of cirrhosis in China at this stage is still viral hepatitis. Although viral hepatitis is currently the primary cause of cirrhosis in China, the proportion of alcoholic cirrhosis has seen an increase in recent years. The epidemiologic study in China is still in the primary stage. The risk of HCC in autoimmune hepatitis is lower than that of other chronic liver diseases. A long-term follow-up study based on 1,428 patients with autoimmune hepatitis (AIH) found that only 1.7% of patients developed HCC with cirrhosis, only mildly increased the risk of HCC (80). Clinical and basic research on autoimmune liver disease in China has just begun, and there is a lack of relevant epidemiologic information, resulting in serious clinical underdiagnosis and mistreatment. In contrast, the evaluation of CHI3L1 in ALD as well as AIH-associated hepatic fibrosis has been reported less frequently, underscoring the need for further exploration of the important role of CHI3L1 in their progression and diagnosis.

## 5 Discussion

The destruction of hepatocytes owing to alcohol abuse, drugs, HBV, and autoimmune factors causes acute injury to the liver. Aseptic inflammation of the liver promotes the repair of liver damage and scar tissue proliferation, overaccumulation of the ECM, and the continuous proliferation of collagenous tissue. If injury factors persist in the liver, the injury can further progresses to hepatic fibrosis, which represents the early stage of a wide range of chronic liver diseases. Liver fibrosis, an early manifestation of various chronic liver diseases, can further develop into cirrhosis or HCC. Early diagnosis and intervention of liver fibrosis are expected to reverse its progression.

Liver tissue biopsy is currently the gold standard for the diagnosis of liver fibrosis; however, its widespread adoption is challenged by its several limitations, including invasiveness, low reproducibility, limitation of sampling, and subjectivity of diagnosis. Current non-invasive tests are mainly divided into imaging and serologic diagnostic models. The LSM detected by FibroScan-based TE can relatively accurately identify progressive liver fibrosis and early cirrhosis, but the measured value is affected by various factors such as liver inflammation and necrosis, cholestasis, and severe steatosis, and there is still a lack of a reliable diagnostic threshold. A serologic diagnosis is more easily accepted as it is more convenient and less influenced by human factors. Currently, the APRI and FIB-4 diagnostic models are acknowledged for their clear diagnostic value. However, both are derived from the data of patients with CHC, and although their diagnostic value in CHB is gradually recognized, the scope of application remains limited. Reliable diagnostic indexes for individual or combined testing have not been identified.

As a biological indicator related to inflammation, CHI3L1 is clinically important for the diagnosis and staging of liver fibrosis caused by various factors, especially for the diagnostic value of advanced liver fibrosis, and it can be used to determine the progression of liver fibrosis.

Serum CHI3L1 is an important diagnostic indicator for CHB liver fibrosis and is important in guiding antiviral therapy. Additionally, CHI3L1 may be associated with insulin resistance and obesity in the pathogenesis of NASH, and hepatic insulin sensitivity can be partially restored by parenteral given anti-Chi3L1 monotherapy (78). Therefore, the development of monoclonal antibody therapeutics targeting CHI3L1 is expected to slow down the progression of NAFLD. Some studies have been conducted or are currently ongoing to investigate the effects of therapy targeting CHI3L1(YKL-40) in the treatment of some medical diseases and various cancer diseases. For example,CHI3L1 inhibitors, including GM-CT-01 and ONO-7475, undergo clinical trials for the treatment of cancer (81). CHI3L1 is also expected to be an independent prognostic factor for HCC. Additionally, significant advances have been made in understanding the pathogenesis of CHI3L1 in liver fibrosis. In hepatitis C, HCV induces and maintains the production of CHI3L1 in liver parenchymal cells by synergistically inducing the TNF-α and ROS-MAPKs pathways through the sustained activation of NF-κB.

As positive feedback, the CHI3L1 protein increases HCV replication and stimulates the release of pro-hepatic fibrotic cytokines and cellular activity in the liver parenchymal cells and HSCs (82). CHI3L1 induces the production of miRNA-449a dysregulation, which regulates CHI3L1 expression by inhibiting the upstream component of the Notch1/NF-κB transcriptional regulatory complex, thereby modulating inflammation. This dysregulation may ultimately lead to liver fibrosis (83).

Thus, CHI3L1 is a very important protein, both as a marker of disease and as a therapeutic target. However, the serum CHI3L1 level alone has little diagnostic value for early liver fibrosis and tends to increase with age. The cellular origin of CHI3L1 in the injured liver and its specific mechanism of action in hepatic fibrosis remain unclear, and more in-depth studies are needed to determine whether CHI3L1 can indeed be used as a therapeutic target.

## Author contributions

XH: Conceptualization, Writing – original draft. WL: Writing – original draft. JL: Writing – review & editing. BW: Writing – original draft. XQ: Conceptualization, Writing – review & editing.
