# Supplementary material for: Research advances in serum chitinase-3-like protein 1 in liver fibrosis
Source: Front Med (Lausanne). 2024 Jun 19;11:1372434. doi: 10.3389/fmed.2024.1372434 (PMC11219575; doi:10.3389/fmed.2024.1372434)
Supplement: Supplementary file 1 [file Data_Sheet_1.PDF]

Supplementary Information for Table2 and Table3

In the Consensus on Diagnosis and Treatment of Liver Fibrosis published in 2019, it is recommended that etiologic and anti-inflammatory hepatoprotective treatments are the mainstay in the early stages of hepatic fibrogenesis, whereas anti-hepatic fibrosis treatments are required in the stage of significant hepatic fibrosis and progression, as well as in the case of cirrhosis.With the growing maturity of non-invasive diagnostic methods for liver fibrosis, serological indicators, liver transient elastography and other imaging techniques can also better reflect the degree of liver fibrosis, and in the Expert Opinion on Expanding Antiviral Therapy for Chronic Hepatitis B issued by the Chinese Medical Association Hepatology Branch in 2022, it is proposed that non-invasive diagnostic hints of obvious inflammation or fibrosis in the liver can be turned on for antiviral therapy.Diagnosing and differentiating the stage of liver fibrosis has important clinical significance for the selection of therapeutic regimens, therefore, in this study, we divided the patients into a significant liver fibrosis group and a non-significant liver fibrosis group according to the liver stiffness value (LSM) of 9.7 kPa, and investigated the diagnostic value of serum CHI3L1 and commonly used non-invasive diagnostic indexes.The study protocol was approved by the Medical Research Ethics Committee of the Second Clinical College of China Medical University [Ethics No. 2023PS947K].

1.1 Subject of the study

(1) Non-alcoholic fatty liver disease(NAFLD)
(2) Chronic hepatitis B (CHB)
(3) Liver cirrhosis
(4) Hepatocellular carcinoma (HCC)

1.2 Inclusion criteria

(1) CHB:According to China's Guidelines for the Prevention and Control of Chronic Hepatitis B (2022 Edition);18 to 65 years of age, HBeAg positive,HBsAg positive,Hepatitis B virus DNA positive for more than 6 months,with or without Hepatitis D Virus Infection,and with chronic liver inflammation.

(2) NAFLD:Diagnostic criteria meet the criteria in the Guidelines for the

Management of Non-Alcoholic Fatty Liver Disease (2018 Update) (soon to be updated to Guidelines for the Management of Metabolism-Related Fatty Liver Disease):1)Liver biopsy specimen showing steatosis (⩾5% of hepatocytes containing fat droplets);2)No history of excessive alcohol consumption in the past 12 months (<30 g/d of ethanol equivalent for men and <20 g/d for women):Exclusion criteria:1)Genotype 3 HCV infection, hepatomegaly, autoimmune hepatitis, total parenteral nutrition,lack of beta lipoproteinemia, congenital lipoatrophy, celiac disease, and other specific diseases that can lead to fatty liver disease:2)Application of drugs such as etofurazone, methotrexate, tamoxifen, glucocorticoids, etc;3)Combination of malignant tumors;4)Combined severe neurological and psychiatric disorders;5)Combined serious diseases of the heart, lungs, kidneys, brain and other important organs.

(3) Liver cirrhosis：Including compensated and decompensated hepatic cirrhosis, it meets the diagnostic criteria for hepatic cirrhosis according to the Guidelines for the Diagnosis and Treatment of Hepatic Cirrhosis.

(4) HCC:HCC can be diagnosed by meeting one of the following three items:1)two typical imaging manifestations of hepatocellular carcinoma (ultrasound, enhanced CT, MRI, or selective hepatic arteriography), with a lesion >2 cm; 2) typical imaging manifestations of hepatocellular carcinoma, with a lesion >2 cm, and an AFP >400 ng/mL; 3) a positive liver biopsy.

1.3 Exclusion criteria

Pregnant women, lactating women and allergic individuals, serious diseases of vital organs, other malignant tumors of the system, autoimmune diseases, as well as acute and chronic infections.

1.4 Data collection

Clinical general information and relevant laboratory parameters: general information such as age, gender, BMI, hypertension, diabetes mellitus, fatty liver, family history, and medication history were collected from the patients;Transient elastography (liver stiffness value LSM + fat attenuation index CAP), liver CT + ultrasound and other imaging data;Liver Fibrosis IV, Liver Function, Renal Function,

Lipids, Glucose, Glycated Hemoglobin, Cardiac Enzyme Profile, Blood Counts, Coagulation Series, Tumor Markers, Ammonia, Rheumatism, Autoantibodies for Liver Disease, and other general labs;Calculation of indicators:APRI,FIB-4，AST/ALT，GGT/ALB，NLR，LMRand other parameters.

1.5 Detection of serum CHI3L1 level by enzyme-linked immunosorbent assay (ELISA)

Fasting venous blood of all subjects was collected in 3 ml, centrifuged at 3500 rpm for 5 minutes and then serum was transferred to a freezing tube and frozen in a -80° C refrigerator, repeated freezing and thawing was prohibited. The serum was equilibrated at room temperature 1 hour before the test. CHI3L1 level was detected by using Shanghai Enzyme-linked Bio Human Chitinase 3-like Protein 1 Assay Kit.

1.6 Histopathologic examination of the liver

Operation method: the patient takes the supine position, under the guidance of B ultrasound, the operator selects the appropriate puncture point and inserts the needle to obtain the biopsy tissue specimen that meets the standard. Experienced pathologists will make histological diagnosis of liver fibrosis by referring to the histopathological staging criteria in the Chinese guidelines for the prevention and treatment of chronic hepatitis B. The diagnosis of liver fibrosis will be confirmed according to the Scheuer scoring system. Liver fibrosis was diagnosed and staged according to the Scheuer scoring system (fibrosis stage ≥S2 was defined as significant liver fibrosis).

**Staging**	**fibrosis**
S0	none
S1	Catchment area enlarged, fibrosis
S2	Dimensional spacer formation, leaflet structure preserved
S3	Fibrous septum with lobular structural disturbances, without cirrhosis
S4	Early cirrhosis or definite cirrhosis

1.7 Transient elastography detection

The FibroTouch test is performed independently by a professionally trained operator with reference to the user manual. The test was performed in the supine

position, with the right hand holding the head to widen the intercostal space, and the detection range was from the right anterior axillary line to the 7th-9th intercostal space in the mid-axillary line, avoiding areas in the liver tissues such as cysts, nodules, and blood vessels that might interfere with the accuracy of the test. At least 10 successful tests were performed in each patient, with a success rate of ≥60% and a detection deviation of <30% of the median value, and the median of the measured values was taken as the final result. Liver stiffness measurement (LSM) and controlled attenuation parameter (CAP) were obtained. Patients were divided into significant liver fibrosis group and no significant liver fibrosis group based on LSM 9.7kPa.

**LSM（kPa）**	**Histopathological staging**
＜7.3	F0-1
7.3~9.7	F2
9.7~12.4	F2-3
12.4~17.5	F3-4
＞17.5	F4

1.8 Statistical methods

For the measurement data, whether the variables obeyed normal distribution was tested by Shapiro-wilk (SW), and $P > 0.05$ indicated that the data obeyed normal distribution, otherwise it was skewed distribution; if it obeyed normal distribution, it was expressed using ($x \pm s$), and the comparison between the two groups was made using the independent samples t-test; otherwise, it was expressed as the median (P50) [interquartile spacing (P25,P75)] Expressed as nonparametric test (Wilcoxon Mann-Whitney two-sample rank-sum test) for comparison between two groups, and Kruskal Wallis test for multi-sample rank-sum test for multiple groups; count data were expressed as the number of cases or percentage, and analyzed by chi-square test. All statistical analyses were performed with $P < 0.05$ as statistically significant differences.

2.Results

2.1 General information

2.1.1 General information on patients with non-viral liver disease

Patients with NAFLD and non-viral hepatitis liver disease were divided into the group without significant liver fibrosis and the group with significant liver fibrosis based on LSM values. There was no statistically significant difference in age, gender, and BMI between the two groups, the percentage of patients with a history of alcohol consumption was higher, PT, PTA, INR, FIB, TT, DD, ALB, PALB, lipase, AFP, CEA, IgA, and IgG differed statistically significantly between the two groups ($P < 0.05$), APTT, GLB, A/G, AST/ALT, PLT, CA199, PLR, SSI differences were statistically significant ($P < 0.001$).

Table 1. General information on patients with non-viral liver disease

	No significant liver fibrosis group(n=30)	Significant liver fibrosis group(n=49)	P-value
Age	49.30±3.00	58.00(47.00,68.50)	0.062
Male（n,%）	15（50）	26(53.1)	0.792
BMI	25.34±0.64	24.49±0.58	0.344
CHI3L1(ng/mL)	113.11(97.56,118.60)	118.49(103.62,128.68)	0.036
APRI	0.45(0.26,0.87)	1.08(0.67,1.93)	<0.001
FIB-4	1.00（0.64，2.37）	4.66(1.95,9.11)	<0.001
Drinking history（n,%）	5(16.7)	23(46.9)	0.006
High blood pressure（n,%）	5(16.7)	13(26.5)	0.310
Diabetes（n,%）	7(23.3)	12(24.5)	0.907
LSM(KPA）	7.60（5.88，8.80）	14.40(11.60,18.20)	<0.001
CAP（db/m）	278.30±6.56	258.59±5.52	0.027
PIIINP	27.40（21.38，35.08）	38.20(24.35,63.35)	0.016

HA	56.58（47.66，67.43）	87.93(56.36,242.80)	0.001
LN	22.86（16.19，27.41）	42.19(23.67,92.08)	<0.001
CIV	24.01（20.83，37.47）	95.13(43.56,171.30)	<0.001
PT	11.10（10.40，11.95）	12.50(11.00,13.85)	0.002
PTA	103.57±3.04	88.63±2.89	0.001
INR	1.00（0.9000，1.03）	1.10(1.00,1.20)	0.002
APTT	31.75±0.46	34.77±0.55	<0.001
FIB	2.82±0.11	2.468±0.08	0.008
TT	18.62±0.19	19.30(18.80,20.15)	0.001
DD	338.70±189.37	111.00(42.00,262.50)	0.037
TP	69.05±0.19	70.83±0.88	0.181
ALB	41.24±1.06	37.53±0.95	0.014
GLB	28（24.90，31.00）	32.20(29.35,36.45)	<0.001
A/G	1.47（1.29，1.75）	1.18(0.97,1.44)	<0.001
AST	32.00（24.50，55.00）	47.00(29.00,92.00)	0.059
ALT	59.50（25.00，97.75）	37.00(19.00,91.00)	0.184
GGT	94.50（36.00，196.50）	93.00(48.50,218.50)	0.712
ALP	93.15（63.05，116.40）	97.70(68.55,139.25)	0.476
PALB	0.25（0.16，0.28）	0.16(0.10,0.21)	0.004
CHE	7420.53±510.84	5613.98±430.06	0.010
TBIL	13.15（10.43，16.60）	19.80(12.35,32.55)	0.011
DBIL	3.85（2.65，5.63）	6.00(3.65,10.55)	0.003
IDBIL	9.25（7.18，10.85）	12.70(8.60,19.60)	0.021
TBA	8.58（5.26，12.94）	17.56(7.33,36.72)	0.003
MAO	5.60（4.71，7.10）	6.01(4.89,8.00)	0.405
AST/ALT	0.56（0.48，1.16）	1.39(0.82,2.00)	<0.001
GGT/ALB	2.45（0.92，4.44）	2.61(1.33,5.18)	0.428
LDL-C	2.92±0.20	2.59±.18	0.249
Lipase	31.30(24.25,41.95)	39.30(29.60,51.05)	0.012
PLT	208.50（159.75，289.25）	116.00(73.00,186.00)	<0.001
PCT	0.21（0.16，0.28）	0.1400(0.0900,0.2350)	0.006
AFP	3.16(2.12,3.92)	4.07(2.73,6.77)	0.048
CEA	1.71（1.14，2.67）	2.70(1.71,3.81)	0.005
CA199	9.71（5.54，15.46）	20.88(12.46,35.19)	<0.001

IgA	2.89(2.14,3.93)	3.70(2.58,6.05)	0.024
IgG	12.15(10.56,14.69)	15.59(12.59,18.87)	0.004
IgM	1.12(0.68,1.49)	1.31(0.93,1.80)	0.064
PLR	118.08(96.95,175.45)	89.38(56.67,115.81)	<0.001
dNLR	-2.27(-2.98，-1.62)	-1.69(-2.85,-1.03)	0.11
SSI	363.02(246.63,541.13)	203.48(125.25,310.52)	<0.001

2.1.3 General information on patients with chronic HBV infection

The two groups were age-matched, with statistically significant differences in gender, HA, LN, INR, CIV, APTT, ALB, GLB, GGT, ALP, TBIL, DBIL, IDBIL, TBA, GGT/ALB, amylase, MCV, AFP, CEA, IgM, and PNI, and statistically significant differences in liver hardness values (KPA), PT, PTA, TT, A/G, CHE, CA199 were statistically different (P < 0.001).

Table 2 General information on patients with chronic HBV infection

	No significant liver fibrosis group(n=45)	Significant liver fibrosis group(n=44)	P-vaue
Age	40.22±1.85	48.32±1.66	0.927
Male（n,%）	26（57.8）	25(56.8)	0.002
CHI3L1(ng/mL)	69.39(45.25，84.88,)	132.51(110.78,157.22)	＜0.001
APRI	0.49(0.29,1.01)	1.02(0.39,2.94)	0.013
FIB4	1.36(0.77,2.14)	2.29(1.46,4.19)	0.001
BMI	23.10(22.10,25.20)	22.95(21.20,25.78)	0.931
Drinking history（n,%）	5（11.1）	8(18.2)	0.345
High blood pressure（n,%）	2（4.4）	3(6.8)	0.627
Diabetes（n,%）	2（4.4）	3(6.8)	0.627
Fatty liver（n,%）	13（28.9）	7（15.9）	0.142
Family history（n,%）	18（40）	15（34.1）	0.564
LSM（KPA）	7.00(6.15,8.30)	13.55(11.45,16.65)	＜0.001

CAP（db/m）	242.00(224.00,275.50)	232.50(221.50,260.00)	0.256
PIIINP	29.70(22.75,37.95)	29.90(23.80,61.30)	0.295
HA	58.11(47.91,66.21)	71.23(53.90,127.48)	0.006
LN	21.94(17.73,26.41	31.14(21.49,60.35)	0.001
CIV	24.53(19.61,37.98)	41.46(23.15,106.51)	0.004
PT	10.90(10.20,11.45)	11.50(11.00,12.15)	＜0.001
PTA	109.00(100.50,119.00)	100.00(91.75,107.00)	＜0.001
INR	1(0.90,1.00)	1.00(1.00,1.08)	0.002
APTT	31.70(30.80,33.05)	33.00(30.90,35.55)	0.025
FIB	2.69(2.41,2.83)	2.50(2.10,2.86)	0.198
TT	18.60(18.00,19.15)	19.35(18.90,19.80)	＜0.001
DD	62.00(20.00,105.00)	63.00(41.00,153.75)	0.231
TP	67.66±0.93	68.64±0.95	0.461
ALB	42.08±0.52	39.01±0.76	0.001
GLB	24.90(21.95,30.90)	28.70(24.93,33.95)	0.002
A/G	1.69(1.41,1.94)	1.39（1.14，1.65）	＜0.001
GGT	28.00(19.00,52.50)	58.00（24.50，125.75）	0.009
ALP	71.90(61.20,82.50)	92.40（69.00，119.78）	0.002
PALB	0.18(0.16,0.22)	0.17（0.11，0.21）	0.102
CHE	8123.00(7059.50,9711.00)	6307.50（3691.00，7416.25）	＜0.001
TBIL	11.5（8.55，15.50）	15.05（11.23，21.60）	0.01
DBIL	3.40（2.05，5.40）	4.25（3.13，7.28）	0.025
IDBIL	8.20（5.90，10.10）	10.10（7.65，14.98）	0.009
TBA	6.00（3.18，8.69）	12.93（5.28，22.77）	0.001
AST/ALT	0.80（0.64，1.16）	0.87（0.68，1.38）	0.412
GGT/ALB	0.70（0.42，1.27）	1.43（0.71，3.46）	0.003
AMY	64.90（48.35，75.65）	73.85（57.20，101.23）	0.032
MCV	87.70（83.75，91.40）	91.40（88.60，94.10）	0.002
AFP	3.18（2.17，3.97）	5.83（2.70，24.93）	0.001
CEA	1.50（1.04，2.23）	1.93（1.41，2.66）	0.041

CA199	9.20（4.17，13.94）	21.74（9.70，45.48）	＜0.001
IgM	49.91±0.84	46.14±0.99	0.005
NLR	1.25(0.79,2.00)	1.57（0.96，2.26）	0.145
LMR	3.96±0.23	4.08±0.26	0.367
PLR	0.06(0.06,0.07)	0.06（0.06，0.07）	0.831
SSI	193.89(105.75,351.19)	200.04(127.09,337.65)	0.806
PNI	49.91±0.84	46.14±0.99	0.005

2.2 Comparison of serum CHI3L1 levels between different liver fibrosis subgroups

In non-CHB patients, the median serum CHI3L1 level was 113.11 (97.56, 118.60) ng/mL in the no-significant liver fibrosis group, and 118.49 (103.62, 128.68) in the significant liver fibrosis group, and there was a statistically significant difference in the distribution of serum CHI3L1 levels between the two groups (z=-2.101 ,P=0.036<0.05); in CHB patients, the median serum CHI3L1 level in the group without significant liver fibrosis was 95.18 (68.39, 112.38) ng/mL, and the median serum CHI3L1 level in the group with significant liver fibrosis was 165.2331 (141.11, 192.65), and the distribution of serum CHI3L1 levels in the two groups were statistically different (z=-7.476,P<0.001).

Table 3. Serum CHI3L1 levels in patients with CHB

Patients with CHB	M (P25, P75)	Median difference （95% CI）	Wilcoxon two-sample rank sum test	
			Z-value	*P*-value
No significant liver fibrosis group (n = 45)	95.18 (68.39–112.38)	-73.489 (-87.320 to -59.509)	-7.476	< 0.001
Significant liver fibrosis group (n = 44)	165.23 (141.11–192.65)			

Table 4. Serum CHI3L1 levels in patients with no hepatitis B

Patients with no CHB	M (P25, P75)	Median difference (95% CI)	Wilcoxon two-sample rank sum test	
			Z-value	*P*-value
No significant liver fibrosis group (n = 30)	113.11 (97.56–118.60)	-8.153 (-16.069 to -0.453)	-2.101	0.036
Significant liver fibrosis group (n = 49)	118.49 (103.62–128.68)			
